# Association between the *FTO *rs9939609 polymorphism and the metabolic syndrome in a non-Caucasian multi-ethnic sample

**DOI:** 10.1186/1475-2840-7-5

**Published:** 2008-03-13

**Authors:** Salam A Al-Attar, Rebecca L Pollex, Matthew R Ban, T Kue Young, Peter Bjerregaard, Sonia S Anand, Salim Yusuf, Bernard Zinman, Stewart B Harris, Anthony JG Hanley, Philip W Connelly, Murray W Huff, Robert A Hegele

**Affiliations:** 1 Vascular Biology Research Group, Robarts Research Institute, London, Ontario, Canada; 2 Department of Public Health Sciences, University of Toronto, Ontario, Canada; 3 National Institute of Public Health, Copenhagen, Denmark; 4 Population Health Research Institute, McMaster University, Hamilton Health Sciences, Hamilton, Ontario, Canada; 5 Department of Medicine, University of Toronto, and Samuel Lunenfeld Research Institute, Mount Sinai Hospital, Toronto, Ontario, Canada; 6 Thames Valley Family Practice Research Unit, University of Western Ontario, London, Ontario, Canada; 7 Department of Nutritional Sciences, University of Toronto, Toronto, Ontario, Canada; 8 Department of Laboratory Medicine and Pathobiology, University of Toronto, and the Keenan Research Centre, Li Ka Shing Knowledge Institute, St. Michael's Hospital, Toronto, Ontario, Canada; 9 Schulich School of Medicine and Dentistry, University of Western Ontario, London, Ontario, Canada

## Abstract

**Background:**

The rs9939609 T>A single-nucleotide polymorphism (SNP) in the *FTO *gene has previously been found to be associated with obesity in European Caucasian samples. The objective of this study is to examine whether this association extends to metabolic syndrome (MetS) and applies in non-Caucasian samples.

**Methods:**

The *FTO *rs9939609 SNP was genotyped in 2121 subjects from four different non-Caucasian geographical ancestries. Subjects were classified for the presence or absence of MetS according to the International Diabetes Federation (IDF) and National Cholesterol Education Program Adult Treatment Panel (NCEP ATP) III definitions.

**Results:**

Carriers of ≥ 1 copy of the rs9939609 A allele were significantly more likely to have IDF-defined MetS (35.8%) than non-carriers (31.2%), corresponding to a carrier odds ratio (OR) of 1.23 (95% confidence interval [CI] 1.01 to 1.50), with a similar trend for the NCEP ATP III-defined MetS. Subgroup analysis showed that the association was particularly strong in men. The association was related to a higher proportion of rs9939609 A allele carriers meeting the waist circumference criterion; a higher proportion also met the HDL cholesterol criterion compared with wild-type homozygotes.

**Conclusion:**

Thus, the *FTO *rs9939609 SNP was associated with an increased risk for MetS in this multi-ethnic sample, confirming that the association extends to non-Caucasian population samples.

## Background

The metabolic syndrome (MetS) is a clinical entity characterized by abdominal obesity, hypertension, hypertriglyceridemia, depressed plasma high-density lipoprotein (HDL) cholesterol and elevated glucose [1,2]. MetS is common, and will likely become even more pervasive, considering the poor lifestyle habits prevalent in many societies today. While the increased prevalence of MetS is primarily related to an imbalance between caloric intake and expenditure, genetic factors are also likely to be important. Each defining component has been previously associated with genetic factors, suggesting that genetic factors might underlie the overall MetS both independently and through more complex interactions [3]. While the precise definition of MetS is controversial, there is no question that the MetS concept has proven to be valuable clinically [4,5].

A potential candidate underlying genetic susceptibility to MetS is the *FTO *(fat mass and obesity associated) gene, encoding the human analogue of *fused toes *in mice. The *FTO *gene encodes a 2-oxoglutarate-dependent nucleic acid demethylase that is present in many tissues, but most abundant in the hypothalamus, the control center of energy balance [6]. Studies in mice showing that *Fto *mRNA levels are regulated by feeding and fasting have provided a mechanistic link between *FTO *and body weight and energy homeostasis [6]. Human population genetic studies have found *FTO *single nucleotide polymorphisms (SNPs) to be associated with type 2 diabetes [7] and obesity [8-11]. The *FTO *rs9939609 SNP is of particular interest since it was found to be associated with obesity through independent studies of large Caucasian populations [8-11]. Since obesity is one of the key features of MetS, we wondered whether this association signal could be found with the complex trait of MetS and whether the association could be detected across other non-Caucasian populations. Thus, the purpose of this study was to examine the *FTO *rs9939609 SNP as a potential genetic candidate for MetS, defined by both the National Cholesterol Education Program Adult Treatment Panel III (NCEP ATP III) and the International Diabetes Federation (IDF) criteria, in a sample derived from multiple non-Caucasian geographic ancestries.

## Methods

### Study subjects

The multi-ethnic study included Canadians of South Asian and Chinese descent, Oji-Cree (Ontario, Canada), and Inuit from Greenland. All subjects for the current study were collected from one of the following population studies: 1) the Study of Health Assessment and Risk in Ethnic Groups [12]; 2) the Sandy Lake Health and Diabetes Project [13]; and 3) the Greenland Population Study [14]. The details of these studies have been described previously [12-14]. Signed informed consent was obtained from all participants and the study was approved by the University of Western Ontario Ethics Review Board (protocol #7920E). For the current analysis, the number of subjects ≥ 18 years of age with both sufficient DNA for *FTO *genotype determination and a complete set of data for MetS diagnosis included 325 South Asians, 299 Chinese, 400 Oji-Cree, and 1097 Greenland Inuit; a total of 2121 subjects (53.9% female). There was no exclusion based on diabetes status.

### Physical measurements

Measurements of waist circumference, blood pressure, fasting analytes, including venous plasma glucose, serum cholesterol, triglycerides (TG), low-density lipoprotein (LDL) cholesterol, and HDL cholesterol were performed as described [12-14].

### Metabolic syndrome classification

According to the NCEP ATP III criteria [1], MetS was identified if a subject had ≥ 3 of: 1) increased waist circumference (>102 cm [>40 inches] for men, >88 cm [>35 inches] for women); 2) elevated plasma TG (≥ 1.69 mmol/L [≥ 150 mg/dL]); 3) low plasma HDL cholesterol (<1.04 mmol/L [<40 mg/dL] for men, <1.29 mmol/L [<50 mg/dL] for women); 4) hypertension (≥ 130/≥ 85 mmHg) or current medication; or 5) impaired fasting glucose (≥ 6.1 mmol/L [≥ 110 mg/dL]).

The IDF criteria [15] identifies MetS for subjects with central obesity, according to ethnic specific guidelines (waist circumference for South Asians and Chinese ≥ 90 cm for men, ≥ 80 cm for women), plus any two of: 1) elevated plasma TG (≥ 1.69 mmol/L [≥ 150 mg/dL]); 2) low plasma HDL cholesterol (<1.04 mmol/L [<40 mg/dL] for men, <1.29 mmol/L [<50 mg/dL] for women); 3) hypertension (≥ 130/≥ 85 mmHg) or current medication; or 4) impaired fasting glucose (≥ 5.6 mmol/L [≥ 100 mg/dL]) or previously diagnosed type 2 diabetes. Since no quantitative thresholds exist yet for aboriginal populations, these subjects were also evaluated using the South Asian and Chinese threshold values for waist circumference.

### Genotyping of the FTO polymorphism

Detection of the *FTO *SNP rs9939609 was carried out using a validated TaqMan genotyping assay (Assay ID C_25638153_10; Applied Biosystems, Foster City, CA). SNP genotyping was performed using an allelic discrimination assay (TaqMan^® ^SNP Genotyping Assays, Applied Biosystems, Foster City, CA) using the 7900HT Fast Real-Time PCR System and genotypes were read using automated software (SDS 2.3, Applied Biosystems, Foster City, CA). Reactions were run in 5 μL volumes using an amplification protocol of 95°C for 10 minutes, followed by 50 cycles of 95°C for 15 seconds, then 60°C for 1.5 minutes.

### Statistical analysis

SAS version 9.1 (SAS Institute, Cary, NC) was used for all statistical comparisons. Data are presented as means ± standard deviation (SD) or as percentages for discrete variables. Between-group differences in discrete variables were analyzed using χ^2 ^analysis and odds ratios (OR) were calculated using the "case-control" method in the FREQ procedure in SAS. Differences in quantitative traits were analyzed by ANOVA, using the general linear model, adjusted for age. The significance of deviations of observed genotype frequencies from those predicted by the Hardy-Weinberg equation were evaluated with χ^2 ^tests. The rs9939609 T>A genotypes were included in the analysis as a dichotomous variable, in both dominant and recessive models. Statistical significance was taken at a P-value<0.05 for all comparisons.

## Results

Table 1 shows the demographic and metabolic characteristics of the populations included in the study. The *FTO *rs9939609 A allele frequencies were 0.32, 0.12, 0.066, and 0.18, for the South Asians, Chinese, Oji-Cree, and Greenland Inuit, respectively. The genotype frequencies did not deviate from the Hardy-Weinberg predictions (data not shown).

**Table 1:** Clinical and biochemical data of subjects

	**Male**	**Female**	***P*-value**
	
**Greenland Inuit**	**n = 486**	**n = 622**	
age (years)	46.2 ± 12.9	45.8 ± 13.1	NS (0.60)
BMI (kg/m^2^)	26.2 ± 4.6	26.7 ± 5.4^2^	NS (0.072)
waist (cm)	91.1 ± 12.2	88.1 ± 13.5	0.0001
systolic BP (mmHg)	120 ± 17	119 ± 20	NS (0.14)
diastolic BP (mmHg)	74 ± 11	72 ± 11	<0.0001
total cholesterol (mmol/L)	5.99 ± 1.18	6.02 ± 1.13	NS (0.77)
triglycerides (mmol/L)	1.15 ± 0.66	1.12 ± 0.61	NS (0.090)
LDL cholesterol (mmol/L)	3.90 ± 1.09^1^	3.89 ± 1.06^2^	NS (0.67)
HDL cholesterol (mmol/L)	1.56 ± 0.49	1.62 ± 0.42	0.0015
fasting glucose (mmol/L)	5.83 ± 0.89	5.76 ± 1.34	NS (0.21)
MetS (%)	12.1	17.0	0.017
			
**Oji-Cree**	**n = 218**	**n = 291**	

age (years)	35.9 ± 14.5	35.7 ± 14.7	NS (0.85)
BMI (kg/m^2^)	26.8 ± 4.6	29.1 ± 5.5	<0.0001
waist (cm)	96.9 ± 12.2	95.0 ± 11.9	NS (0.073)
systolic BP (mmHg)	122 ± 14	118 ± 16	<0.0001
diastolic BP (mmHg)	70 ± 12	67 ± 10	<0.0001
total cholesterol (mmol/L)	4.83 ± 0.98	4.59 ± 0.82	0.0001
triglycerides (mmol/L)	1.63 ± 0.85	1.51 ± 0.71	0.0003
LDL cholesterol (mmol/L)	2.91 ± 0.82^3^	2.62 ± 0.65^4^	<0.0001
HDL cholesterol (mmol/L)	1.19 ± 0.30	1.28 ± 0.28	<0.0001
fasting glucose (mmol/L)	6.73 ± 3.25	6.77 ± 3.50	NS (0.61)
MetS (%)	30.3	39.2	0.028
			
**South Asian**	**n = 180**	**n = 147**	

age (years)	49.9 ± 8.9	49.0 ± 9.6	NS (0.37)
BMI (kg/m^2^)	26.1 ± 4.3^5^	26.5 ± 3.8	NS (0.37)
waist (cm)	94.7 ± 9.7	86.0 ± 10.6	<0.0001
systolic BP (mmHg)	121 ± 16	117 ± 19	0.034
diastolic BP (mmHg)	80 ± 12	71 ± 10	<0.0001
total cholesterol (mmol/L)	5.26 ± 0.98	5.15 ± 0.94	NS (0.41)
triglycerides (mmol/L)	2.05 ± 1.31	1.92 ± 1.27	NS (0.30)
LDL cholesterol (mmol/L)	3.45 ± 0.84^6^	3.18 ± 0.79^7^	0.0069
HDL cholesterol (mmol/L)	0.95 ± 0.26	1.13 ± 0.32	<0.0001
fasting glucose (mmol/L)	5.95 ± 1.89	5.49 ± 1.58	0.020
MetS (%)	31.1	34.0	NS (0.38)
			
**Chinese**	**n = 148**	**n = 151**	

age (years)	48.8 ± 9.3	46.6 ± 8.4	0.030
BMI (kg/m^2^)	25.2 ± 3.4	22.7 ± 3.5	<0.0001
waist (cm)	89.6 ± 9.1	75.2 ± 7.9	<0.0001
systolic BP (mmHg)	122 ± 17	112 ± 20	0.022
diastolic BP (mmHg)	80 ± 10	70 ± 12	<0.0001
total cholesterol (mmol/L)	5.29 ± 1.00	4.85 ± 0.84	0.0057
triglycerides (mmol/L)	2.14 ± 1.61	1.38 ± 1.03	0.0055
LDL cholesterol (mmol/L)	3.34 ± 0.84^8^	2.90 ± 0.71^9^	0.0006
HDL cholesterol (mmol/L)	1.03 ± 0.29	1.33 ± 0.36	<0.0001
fasting glucose (mmol/L)	5.44 ± 1.29	4.98 ± 0.60	0.016
MetS (%)	28.4	10.6	0.0003

^1^n = 483, ^2^n = 620, ^3^n = 216, ^4^n = 289, ^5^n = 179, ^6^n = 169, ^7^n = 141, ^8^n = 139, ^9^n = 148

Abbreviations: BMI, body mass index; BP, blood pressure; LDL, low-density lipoprotein; HDL, high-density lipoprotein; MetS, metabolic syndrome; NS, not significant.

Data are means ± standard deviation (SD). *P*-values are adjusted for age; *P*-values for blood pressure, cholesterol, triglycerides, LDL cholesterol, HDL cholesterol, and glucose are also adjusted for BMI.

Analysis across the 4 study populations indicated that *FTO *rs9939609 A allele carriers had an increased risk of MetS, according to the IDF definition (carrier OR 1.23, 95% CI 1.01 to 1.50, *P *= 0.036) (Table 2). Subgroup analysis of females showed that the carrier OR was 1.05 (95% CI 0.80 to 1.38, *P *= NS [0.74]). Subgroup analysis of males showed that the carrier OR was 1.46 (95% CI 1.10 to 1.94, *P *= 0.0081). By individual ethnic populations, a significant increased risk for MetS was observed for *FTO *rs9939609 A allele carriers only in the South Asian group (*P *= 0.027). Analysis of the NCEP ATP III definition for MetS showed a similar trend towards increased risk for *FTO *rs9939609 A allele carriers compared to TT homozygotes (carrier OR 1.26, 95% CI 1.02 to 1.57, *P *= 0.036) (Table 2). A significant increased risk for NCEP ATP III MetS was observed for *FTO *rs9939609 A allele carriers among Greenland Inuit (*P *= 0.037).

**Table 2:** Metabolic syndrome and metabolic syndrome component prevalence in subjects when classified in accordance to their genotype of the *FTO *rs9939609 T>A polymorphism

	**TT**	**AA & AT**	**OR (95% CI)**	**P-value**
Overall N = 2121	1482	639		
				
IDF MetS (%)	31.2	35.8	1.23 (1.01, 1.50)	0.036

South Asian	36.8	49.1	1.66 (1.06, 2.58)	0.027
Chinese	24.6	22.4	0.89 (0.46, 1.69)	0.71
Oji-Cree	48.9	51.9	1.13 (0.63, 2.03)	0.68
Greenland Inuit	23.9	29.4	1.33 (0.99, 1.77)	0.051
				
NCEP ATP III MetS (%)	21.1	25.2	1.26 (1.02, 1.57)	0.036

South Asian	28.3	35.8	1.42 (0.88, 2.27)	0.15
Chinese	18.5	22.4	1.27 (0.65, 2.46)	0.48
Oji-Cree	36.2	40.4	1.19 (0.66, 2.17)	0.56
Greenland Inuit	13.3	18.2	1.44 (1.02, 2.04)	0.037
				
MetS BP (%)	30.3	33.2	1.14 (0.94, 1.39)	0.19

South Asian	35.5	33.0	0.89 (0.56, 1.41)	0.62
Chinese	32.8	40.3	1.39 (0.79, 2.43)	0.25
Oji-Cree	27.6	19.2	0.63 (0.30, 1.30)	0.20
Greenland Inuit	29.7	34.0	1.22 (0.93, 1.60)	0.16
				
MetS Triglycerides (%)	25.0	26.5	1.08 (0.87, 1.34)	0.47

South Asian	46.1	46.8	1.03 (0.67, 1.60)	0.89
Chinese	38.8	31.3	0.72 (0.40, 1.29)	0.27
Oji-Cree	33.1	40.4	1.37 (0.76, 2.49)	0.30
Greenland Inuit	12.7	13.3	1.05 (0.72, 1.54)	0.79
				
MetS HDL (%)	33.7	39.6	1.29 (1.07, 1.56)	0.0089

South Asian	69.1	71.7	1.13 (0.70, 1.83)	0.61
Chinese	51.7	61.2	1.47 (0.85, 2.56)	0.17
Oji-Cree	45.7	59.6	1.75 (0.97, 3.17)	0.061
Greenland Inuit	15.3	16.4	1.09 (0.77, 1.53)	0.64
				
MetS Glucose (%)	45.3	47.7	1.10 (0.92, 1.33)	0.30

South Asian	25.7	34.1	1.50 (0.93, 2.43)	0.098
Chinese	17.2	20.9	1.27 (0.64, 2.50)	0.49
Oji-Cree	53.5	44.2	0.69 (0.38, 1.24)	0.21
Greenland Inuit	54.1	60.2	1.28 (0.99, 1.66)	0.059
				
MetS Waist (IDF) (%)	60.9	65.0	1.19 (0.98, 1.44)	0.080

South Asian	64.5	75.1	1.67 (1.03, 2.69)	0.036
Chinese	34.9	32.8	0.91 (0.51, 1.62)	0.75
Oji-Cree	81.0	78.9	0.87 (0.43, 1.79)	0.71
Greenland Inuit	58.9	64.0	1.24 (0.95, 1.61)	0.11
				
BMI>33 kg/m^2 ^(%)	8.1	10.6	1.35 (0.99, 1.84)	0.060

South Asian	4.0	5.8	1.48 (0.53, 4.18)	0.45
Chinese	2.2	1.5	0.69 (0.079, 5.99)	0.73
Oji-Cree	13.5	26.9	2.36 (1.19, 4.68)	0.012
Greenland Inuit	8.3	12.4	1.57 (1.04, 2.36)	0.032

No significant increased risk of MetS was observed overall for AA homozygotes, for either the IDF or NCEP ATP III definitions, upon repeating the analyses using a recessive model for the *FTO *rs9939609 A allele. The non-significant trends suggested that sample numbers were insufficient for detecting an association with this particular trait.

Analysis of the entire sample revealed no significant increase in blood pressure, plasma triglycerides or glucose levels (Table 2). However, meta-analysis indicated that significantly more *FTO *rs9939609 A allele carriers met the MetS criteria for depressed HDL cholesterol (*P *= 0.0089), and there was a nonsignificant trend towards increased waist circumference and increased BMI for *FTO *rs9939609 A allele carriers (*P *= 0.080 and 0.060, respectively) (Table 2).

## Discussion

Our study of *FTO *as a candidate gene for MetS in a sample from multiple non-Caucasian geographical ancestries showed 1) significant association with the *FTO *gene, with rs9939609 A allele carriers, particularly males, having an increased risk of MetS (carrier OR 1.23, 95% CI 1.01 to 1.50; *P *= 0.036) (Table 2); and 2) this association was related to higher proportion of subjects with depressed HDL cholesterol and a trend towards increased waist circumference. Similar trends were observed for the NCEP ATP III definition of MetS.

This is the first report of an association between the *FTO *rs9939609 A allele and MetS. Furthermore, it extends the association of this allele beyond Caucasian samples. Although the observation may help understand the molecular etiology of MetS, this association requires replication in larger studies, considering the possibility of a spurious association due to population stratification or another such limitation [16]. Despite considerations regarding sample size however, the results reaffirm the established association between the *FTO *rs9939609 A allele with obesity in past findings [8-11]. However, MetS is a complex disorder, and only a few of the previously associated genes, such as *APOC3 *and *PPARG*, have been replicated in more than one study sample [4]. Aside from the need to replicate the *FTO *association in other larger study samples, the mechanism underlying the association also needs to be investigated. The mechanism underlying the association may be related to: 1) a direct function of *FTO *[6] or 2) linkage disequilibrium of the tested variant with another causative change in a gene near the *FTO *locus.

Analysis of the overall sample indicated that significantly more *FTO *rs9939609 A allele carriers met the MetS criteria for depressed HDL cholesterol (*P *= 0.0089) than non-carriers. In addition, values were close to significance also for increased waist circumference (*P *= 0.080) and increased BMI (*P *= 0.060) among A allele carriers (Table 2). This indicates that the association of the *FTO *rs9939609 A allele with the MetS was related in part to both the obesity component, but also the HDL component, suggesting that this genetic marker may have a broader relationship with individual components of this complex trait.

Although the meta-analysis across the four non-Caucasian study populations showed an association between MetS and the common rs9939609 SNP in *FTO*, analyses by individual ethnic populations indicated a significant increased risk for MetS only among the South Asians (IDF definition) and Greenland Inuit (NCEP ATP III definition). Thus, the overall association is primarily due to these two populations, with little influence from the Chinese and Oji-Cree. Again, replication of the *FTO *association in other larger study samples is essential for clarification. Further evidence against an association for the Chinese can be found in a recent study of 3,210 unrelated Chinese Han subjects from Shanghai and Beijing where no association found between the rs9939609 SNP and obesity [17].

In summary, we report an association between MetS and the common rs9939609 SNP in *FTO*. The observed consistent association between MetS and *FTO *in a non-Caucasian multi-ethnic study group, including populations which differ considerably in MetS prevalence, strengthens the likelihood that the *FTO *locus is related to obesity and MetS. Future replication of this association in larger sized samples and devising functional molecular studies will further enhance the validity of association and the causative relationship between the *FTO *variant and MetS.

## Competing interests

The author(s) declare that they have no competing interests.

## Authors' contributions

SAA and RLP participated in the experimental design, data acquisition and analysis, interpretation of results, and manuscript writing. MRB participated in the analysis of the data. TKY, PB, SSA, SY, BZ, SBH, AJGH, PWC, and MWH were involved in the provision of patient samples and/or clinical data. RAH participated in the experimental design, data analysis and interpretation of results and manuscript writing. All authors approved the final manuscript.
